# Connecting care—telemedicine breaks barriers for low-income Brazilians battling breast cancer: a prospective cohort study

**DOI:** 10.1590/1516-3180.2025.3116.09032026

**Published:** 2026-06-15

**Authors:** Gabriel Salum D’Alessandro, Yedda Nunes Reis, Mila Trementosa Garcia, Daniela Zaros Guimarães, Roberta Amparado Miziara, Flavia Colsomagno Silveira Berlofa, Mayara de Cássia Benedito Andrade, Emily Rie Yoshizato, Jonathan Yugo Maesaka

**Affiliations:** I Hospital Israelita Albert Einstein, São Paulo (SP), Brazil.; II Hospital Israelita Albert Einstein, São Paulo (SP), Brazil.; III Hospital Israelita Albert Einstein, São Paulo (SP), Brazil.; IV Hospital Israelita Albert Einstein, São Paulo (SP), Brazil.; V Hospital Israelita Albert Einstein, São Paulo (SP), Brazil.; VI Hospital Israelita Albert Einstein, São Paulo (SP), Brazil.; VII Hospital Israelita Albert Einstein, São Paulo (SP), Brazil.; VIII Albert Einstein Medical College, São Paulo (SP), Brazil.; IX Hospital Israelita Albert Einstein, São Paulo (SP), Brazil.

**Keywords:** Telemedicine, Breast neoplasms, Developing countries, Healthcare disparities, Health equity, Breast cancer, Healthcare access, Low-income population, Tele-oncology, Patient satisfaction, Public health

## Abstract

**BACKGROUND:**

Telemedicine may help advance healthcare equity, particularly in low-income communities, by reducing economic burdens and mitigating geographic barriers to specialized care, including transportation costs, time away from work, and long travel distances to referral centers.

**OBJECTIVES:**

This study aims to evaluate the impact of a remote consultation protocol on healthcare access among predominantly low-income patients with breast cancer in Brazil.

**DESIGN AND SETTING:**

This prospective cohort study was conducted at a tertiary public cancer center and included 946 women with breast cancer.

**METHODS:**

Sociodemographic characteristics, teleconsultation-related data, and patient satisfaction measured using a validated Likert-based questionnaire were collected. The study was approved by the local ethics committee, and all participants provided informed consent.

**RESULTS:**

The mean age was 57.3 years. Overall, 51.6% of participants identified as white, and 64.4% reported a family income below two Brazilian minimum wages. Over one-third (34.3%) had not completed elementary school. Despite this socioeconomic vulnerability, 93.5% reported internet access, 90.3% owned a mobile phone, and 52.7% had an email address. During the study period, 946 appointments were scheduled and screened for teleconsultation eligibility. Among these, 229 patients met predefined eligibility criteria and were offered teleconsultation, and 117 completed the remote visit, corresponding to an uptake of 51.1% among eligible patients. Regarding hospital visits, 57% relied on public transportation, with a mean cost of $ 4 per visit, representing a substantial financial burden. Travel time exceeded 90 minutes for 19.4% of patients. Satisfaction with teleconsultation was high, with a mean Likert score of 4.65/5 and a Net Promoter Score of 79.8.

**CONCLUSION:**

Among patients eligible for telemedicine, more than half completed teleconsultation, indicating that implementation is feasible even in socially vulnerable populations with limited digital literacy. The remote care protocol improved access, reduced financial burden, and achieved high patient satisfaction, supporting telemedicine as a strategy to promote equity in breast cancer care in Brazil.

## INTRODUCTION

Achieving equitable healthcare remains a major global challenge, particularly in predominantly low-income communities, where economic and geographic barriers impede access to medical services.^1^ Telemedicine has emerged as a promising approach to address these inequities by enabling remote access to healthcare professionals and potentially improving the inclusivity and accessibility of care, especially for patients undergoing breast cancer treatment.^2^

As an important component of medical informatics, telemedicine uses information and communication technologies to deliver healthcare in a more cost-effective, efficient, and accessible manner, particularly in economically disadvantaged settings.^3,4^ Evidence indicates that telemedicine can be especially beneficial for vulnerable populations, including older adults, individuals with limited mobility, and ethnic minorities, thereby helping to reduce disparities in access to care.^5^

Equity in healthcare is grounded in the principle that individuals who face greater barriers to accessing services should receive additional support to achieve comparable access, treatment, and outcomes.^6^ In this context, strengthening healthcare delivery for socioeconomically disadvantaged populations is essential to improving health outcomes and advancing broader health system objectives.^7,8^

In Brazil, breast cancer represents a major public health concern and disproportionately affects vulnerable populations with limited access to healthcare services. This limited access creates important challenges and potential risks to optimal patient management across a prolonged and complex treatment pathway.^9^ Telemedicine has emerged as a potential strategy to mitigate these difficulties by providing remote clinical support and reducing the financial and time burdens associated with in-person care.^10^

Territorial disparities in the distribution of healthcare services have long affected access to care in Brazil, reinforcing the need for context-specific strategies and policy solutions.^11,12^ In response to these challenges, some public healthcare facilities in Brazil have begun integrating telemedicine into the national public health system as a means of expanding access and addressing persistent inequities.^1,13,14,15^

This study evaluated the impact of a telemedicine protocol for remote consultations on healthcare equity among a predominantly low-income population of patients with breast cancer treated at a Brazilian public hospital. Specifically, by implementing telephone-based telemedicine consultations, the study examined their effects on patient satisfaction and access to care.

## OBJECTIVE

The primary objective was to assess patient satisfaction with remote care provided by the breast surgery team using a telemedicine satisfaction questionnaire. The secondary objectives included evaluating the socioeconomic profile of participating patients, identify the reasons for eligibility for remote care, and determine the proportion of scheduled outpatient appointments considered eligible for telemedicine.

## METHODS

### Study design and population

This prospective cohort study evaluated the implementation of telemedicine in the clinical practice of a breast surgery team at a municipal public hospital in São Paulo, Brazil, between July 2023 and April 2024. The study was approved by the institutional Research Ethics Committee (Approval No. CAAE 68518523.0.0000.0071) and was conducted in accordance with Resolution No. 466/12 of the Brazilian National Health Council. Written informed consent was obtained from all participants before enrollment. Consent was obtained during a prior inperson visit to enable subsequent teleconsultation. The study was reported in accordance with the STROBE guidelines for observational studies.^16^

Eligible participants were adult patients aged **≥** 18 years attending the breast surgery outpatient clinic. Although men were eligible for inclusion, no male patients were enrolled in the study.

As a public tertiary care service, the hospital did not provide screening consultations or second opinions. All participants had a confirmed diagnosis before referral and were managed within a predefined treatment pathway.

Patients undergoing neoadjuvant systemic therapy under shared follow-up by the clinical oncology and breast surgery teams were eligible for inclusion. During neoadjuvant treatment, surgical visits were scheduled for predefined purposes, including review of treatment response based on imaging findings and oncology reports, surgical planning, coordination of multidisciplinary care, and patient counseling. These visits did not involve physical examination, diagnostic assessment, or delivery of sensitive results. Accordingly, this neoadjuvant subgroup comprised established patients at intermediate to late stages of a predefined treatment pathway.

Exclusion criteria included any condition requiring in-person evaluation, such as clinical instability, need for physical examination (e.g., new breast complaints), pending diagnostic imaging or laboratory results requiring face-to-face discussion, or situations involving sensitive communication, such as suspected disease recurrence or disclosure of unfavorable diagnostic findings. Early postoperative follow-up visits were excluded by design because they routinely require physical examination. Patients attending their first consultation with the breast surgery team were also excluded. Additional exclusion criteria were significant cognitive impairment without a legal guardian and refusal to provide informed consent.

For participant recruitment, the outpatient schedule was reviewed weekly to identify patients eligible for remote care. Eligible patients were contacted by telephone at the same day and time as their originally scheduled in-person consultation and were offered teleconsultation. Following the teleconsultation, participants completed the Telemedicine Satisfaction Questionnaire (TSQ), which had been translated and validated for use in Brazil.^17^ This questionnaire comprises 14 items addressing multiple aspects of remote care.

### Data collection and definitions

All data were collected, securely stored, and extracted using the REDCap electronic data capture platform.^18^ Anonymization procedures were rigorously applied to protect patient confidentiality.

### Statistical analysis

Descriptive analyses were performed using absolute frequencies and percentages for categorical variables. Numerical variables were summarized using means and standard deviations, medians and quartiles, and minimum and maximum values, as appropriate. The distributions of numerical variables were assessed using histograms, boxplots, quantile comparison plots, and the Shapiro–Wilk normality test.

Differences in satisfaction scores between the first and second teleconsultations were assessed using the Wilcoxon signed-rank test for paired data. Associations between categorical variables were examined using the chi-square test. Relationships between patients’ sociodemographic characteristics and scores on the Telemedicine Patient Satisfaction Assessment Questionnaire were evaluated using the Mann-Whitney test or Kruskal-Wallis test for categorical variables and Spearman correlation coefficients for numerical variables.

All analyses were performed using IBM SPSS Statistics for Windows version 29.0.2022 (IBM Corp., Armonk, New York)., with a two-sided significance level of 5%.

## RESULTS

**Figure 1:**
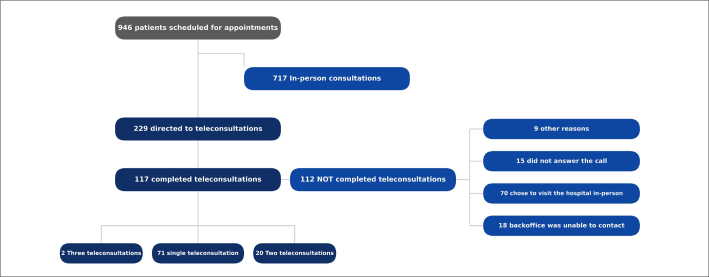
Flow-chart of the study.

During the study period, a total of 946 patients were scheduled for appointments at the breast surgery clinic. All scheduled visits were screened for teleconsultation eligibility according to predefined criteria. Of these, 229 patients met the eligibility criteria and were offered teleconsultation. Among the eligible patients, 117 (51.1%) completed the teleconsultation, whereas 112 did not. Reasons for non-completion included failure of the back-office team to establish contact (n = 18), unanswered calls (n = 15), patient preference for an in-person consultation (n = 70), and other reasons (n = 9) (**Figure 1**).

Over the 43-week study period, 117 teleconsultations were conducted. Of the patients who received remote care, 71 had one teleconsultation, 20 had two, and two had three teleconsultations. Among the 22 patients who received two remote consultations, the interval between appointments ranged from 14 to 301 days, with a median of 46 days. For the two patients who underwent three teleconsultations, the interval between the second and third consultations was 70 days for one patient, and 126 days for the other.

The main reasons for referral to remote care were the unavailability of a relevant examination required for decision-making (40.2%) and ongoing neoadjuvant chemotherapy with active follow-up by the clinical oncology team (29.9%).

Based on the first teleconsultation for each patient, the sociodemographic characteristics and teleconsultation-related data of 93 patients are presented in **Tables 1 and 2**.

**Table 1:** Socioeconomic characteristics (n = 93)

Variable	Category	n (%)
Age (years)	Mean (SD)	57.3 (13.8)
	Median (Q1; Q3)	57 (46; 67)
	Minimum; maximum	29; 86
Ethnicity	White	50 (53.8%)
	Black	9 (9.7%)
	Brown	31 (33.3%)
	Asian	3 (3.2%)
Marital Status	Single	23 (24.7%)
	Married	33 (35.5%)
	Divorced	14 (15.1%)
	Widowed	11 (11.8%)
	Stable union/Cohabitating	12 (12.9%)
Education Level	No formal education	6 (6.5%)
	Incomplete elementary education	32 (34.4%)
	Complete elementary education	16 (17.2%)
	Incomplete high school education	6 (6.5%)
	Complete high school education	21 (22.6%)
	Incomplete higher education	3 (3.2%)
	Complete higher education	9 (9.7%)
Monthly Family	Up to two minimum wages	62 (66.7%)
Income^*^	More than two minimum wages	31 (33.3%)
Work Status	Unemployed	36 (38.7%)
	Formal/informal work	21 (22.6%)
	Government assistance income	36 (38.7%)

SD, standard deviation; Q1, first quartile; Q3, third quartile;

* During the study period (July 2023–April 2024), the national minimum wage was R$ 1,320 in 2023 and R$ 1,412 in 2024 (approximately $ 250–270 per month, depending on exchange rates).

**Table 2:** Teleconsultation-related data (n = 93)

Variable	Category	n (%)
Teleconsultation	No	13 (61.9%)
prevented workday loss^*^	Yes	8 (38.1%)
Internet access	No	6 (6.5%)
	Yes	87 (93.5%)
Cell phone ownership	No	9 (9.7%)
	Yes	84 (90.3%)
Email ownership	No	44 (47.3%)
	Yes	49 (52.7%)
Distance from residence to hospital (km)	Mean (SD)	12.8 (7.9)
	Median (Q1; Q3)	13 (5.6; 18)
	Minimum; maximum	0.6; 39
Mode of transport to the hospital	Public transport	53 (57%)
	Private car	28 (30.1%)
	Taxi/app-based driver	11 (11.8%)
	On foot	1 (1.1%)
Transportation cost (round-trip)^**^	Up to $ 2	33 (35.5%)
	$ 2–4	25 (26.9%)
	$ 4–10	20 (21.5%)
	More tan $ 10	15 (16.1%)
Travel time (one way) to the hospital	Up to 30 minutes	18 (19.4%)
	30–45 minutes	16 (17.2%)
	45 minutes–1 hour	16 (17.2%)
	1–1.5 hours	17 (18.3%)
	> 1.5 hours	26 (28%)
Attends consultations with a companion	Alone	20 (21.5%)
	With a companion	73 (78.5%)
Reason for referral to teleconsultation	Unavailable exam^***^	47 (40.2%)
	Undergoing neoadjuvant chemotherapy	35 (29.9%)
	Unperformed interconsultation	15 (12.8%)
	Only for exam request	9 (7.7%)
	Check test results	8 (6.8%)
	Incorrect outpatient scheduling	1 (0.9%)
	Other	2 (1.7%)

SD, standard deviation; Q1, first quartile; Q3, third quartile;

* Only those employed (n = 21);

** At the time of analysis, during July 2024, the US dollar exchange rate fluctuated between approximately R$ 5.40 and R$ 5.50;

*** e.g., missed imaging appointment, delayed results, test not performed, or results unavailable in the system at the time of the visit.

The mean age was 57.3 years. Most patients were white, married, and had not completed elementary school (**Table 1**). Overall, 66.7% reported a monthly family income of up to two minimum wages, 38.7% were unemployed, and another 38.7% were dependent on government assistance. Among patients engaged in formal or informal employment, 38.1% reported avoiding the loss of a workday because of remote care (**Tables 1 and 2**).

Regarding digital connectivity, 93.5% reported having internet access, 90.3% owned a mobile phone, and 52.7% had a personal email account (**Table 2**).

The mean distance from the patient’s residence to the hospital was 12.8 km. Most patients relied on public transportation, spent up to $ 2 to reach the hospital, and required over 90 minutes for travel, often accompanied by another person during consultations (**Table 2**).

**Figure 2:**
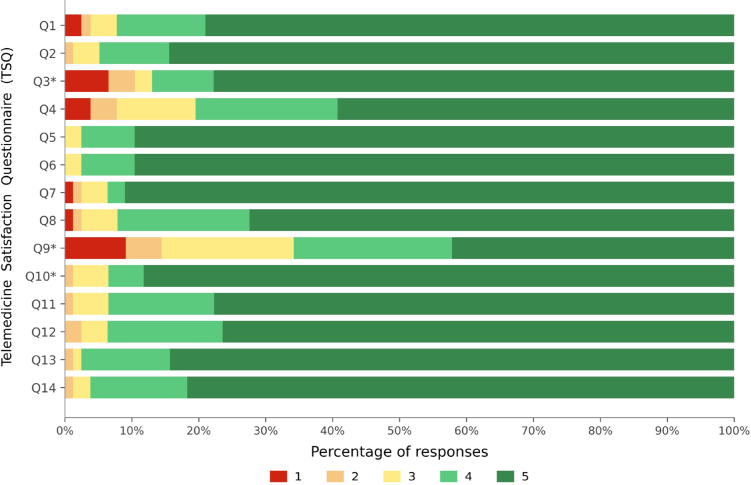
Distribution of scores for questions answered by patients on the TSQ instrument (n = 94).

The Patient Satisfaction Assessment Questionnaire for Telemedicine was completed in 94 of the 117 teleconsultations (80.3%). Among these, 76 questionnaires were completed after the first teleconsultation, 16 after the second, and two after the third. TSQ scores ranged from 2.4 to 5.0, with a median of 4.8 (**Tables 3, 4, and Figure 2**).

**Table 3:** Distribution of patient responses on the TSQ instrument regarding teleconsultations (n = 94)

Questions	Responses				
	No, definitely not	Probably not	Maybe	Probably yes	Yes, for sure
**Q1. Telemedicine is better than I expected.**	2 (2.1%)	1 (1.1%)	3 (3.2%)	12 (12.8%)	76 (80.9%)
**Q2. I am satisfied with my telemedicine care.**	0 (0%)	1 (1.1%)	3 (3.2%)	12 (12.8%)	78 (83%)
**Q3. I was worried about my privacy during the telemedicine consultation.^#^**	76 (80.9%)	7 (7.4%)	2 (2.1%)	3 (3.2%)	6 (6.4%)
**Q4. The care I received by telemedicine was as good as inperson care.**	3 (3.2%)	4 (4.3%)	11 (11.7%)	19 (20.2%)	57 (60.6%)
**Q5. Telemedicine saved me travel time.**	0 (0%)	0 (0%)	2 (2.1%)	7 (7.4%)	85 (90.4%)
**Q6. Telemedicine saved me money.**	0 (0%)	0 (0%)	3 (3.2%)	7 (7.4%)	84 (89.4%)
**Q7. I felt comfortable talking to the doctor on the phone.**	1 (1.1%)	1 (1.1%)	4 (4.3%)	2 (2.1%)	86 (91.5%)
**Q8. I felt that my care was complete.**	1 (1.1%)	2 (2.1%)	5 (5.3%)	17 (18.1%)	69 (73.4%)
**Q9. I prefer spending more time traveling for my next appointment in person rather than telemedicine.^#^**	39 (41.5%)	23 (24.5%)	20 (21.3%)	4 (4.3%)	8 (8.5%)
**Q10. I had difficulty hearing the doctor on the phone.^#^**	83 (88.3%)	4 (4.3%)	4 (4.3%)	2 (2.1%)	1 (1.1%)
**Q11. I was able to build a trusting relationship with the doctor.**	0 (0%)	1 (1.1%)	6 (6.4%)	13 (13.8%)	74 (78.7%)
**Q12. I was able to explain my problems clearly to the doctor during telemedicine care.**	0 (0%)	2 (2.1%)	3 (3.2%)	15 (16%)	74 (78.7%)
**Q13. Telemedicine care was good for me.**	0 (0%)	1 (1.1%)	1 (1.1%)	12 (12.8%)	80 (85.1%)
**Q14. I would recommend telemedicine care to other patients (NPS).**	0 (0%)	1 (1.1%)	2 (2.1%)	13 (13.8%)	78 (83%)

^#^ Questions with negatively worded statements.

**Table 4:** Description of Results from the Application of the Patient TSQ for Telemedicine Consultations (n=94)

Questions from the instrument	Mean (SD)
**Q1**	4.69 (0.78)
**Q2**	4.78 (0.55)
**Q3**	4.53 (1.12)
**Q4**	4.31 (1.05)
**Q5**	4.88 (0.38)
**Q6**	4.86 (0.43)
**Q7**	4.82 (0.66)
**Q8**	4.61 (0.78)
**Q9**	3.86 (1.25)
**Q10**	4.77 (0.72)
**Q11**	4.70 (0.64)
**Q12**	4.71 (0.63)
**Q13**	4.82 (0.49)
**Q14**	4.79 (0.53)
**Instrument score**	
**Mean (SD)**	4.65 (0.43)
**Median (Q1; Q3)**	4.79 (4.43; 5)
**Minimum; maximum**	2.36; 5

SD: Standard deviation; Q1: First quartile; Q3: Third quartile

* Questions with negatively worded statements.

Based on the isolated response to question 14, “I would recommend telemedicine to other patients,” the Net Promoter Score (NPS) was calculated to assess satisfaction with teleconsultation. The NPS was defined as the percentage of promoters minus the percentage of detractors, with scores between 75% and 100% generally considered excellent. Among the 94 questionnaires, 3.2% of respondents were classified as detractors and 83% as promoters, resulting in an NPS of 79.8%.

**Figure 3:**
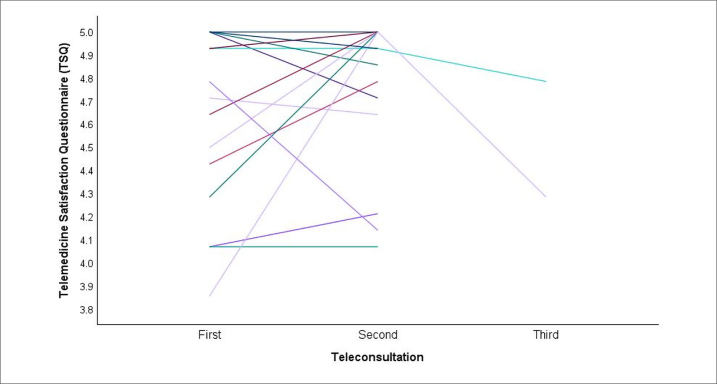
Individual TSQ scores of patients who underwent more than one teleconsultation (n = 15).

Patients who underwent more than one teleconsultation could complete the TSQ after each appointment. Fifteen patients completed the questionnaire twice, including the two patients who underwent three teleconsultations and responded after each visit. When satisfaction scores from the first and second teleconsultations were compared, seven patients had higher scores after the second consultation, five had lower scores, and three had unchanged scores (**Figure 3**). After the first teleconsultation, scores ranged from 3.86 to 5.00, with a median of 4.71 (first quartile, 4.29; third quartile, 5). After the second teleconsultation, scores ranged from 4.07 to 5.00, with a median of 4.93 (first quartile, 4.64; third quartile, 5).No significant difference was observed between the first and second teleconsultation scores (p = 0.254).

Additionally, no significant differences were found in the distribution of TSQ scores according to age group (p = 0.861), educational level (p = 0.530), monthly family income (p = 0.242), employment status (p = 0.832), transportation cost to the hospital (p = 0.762), or travel time to the hospital (p = 0.287). In addition, weak and non-significant correlations were observed between TSQ score and patient age (correlation coefficient = 0.024; p = 0.839) and between TSQ score and distance from residence to hospital (correlation coefficient = 0.123; p = 0.291) (**Table 5**).

**Table 5:** Associations between sociodemographic data and TSQ after the first teleconsultation (n = 76)

Variable	Median (Q1; Q3)	Min; max	n	p value
Age (years)				
≤ *39*	4.50 (4.14; 5)	3.93; 5	7	
*40–49*	4.93 (4.21; 5)	2.36; 5	15	0.861^b^
*50–60*	4.79 (4.57; 4.93)	3.86; 5	27	
≥ *61*	4.79 (4.43; 5)	3.64; 5	27	
Education				
*No schooling to primary*	4.79 (4.54; 5)	3.79; 5	40	
*Incomplete/complete high school*	4.79 (4.14; 5)	3.64; 5	25	0.53^b^
*Incomplete/complete higher education*	4.71 (4.43; 4.93)	2.36; 4.93	11	
Monthly family income (IBGE)				
*Up to 2 minimum wages*	4.79 (4.25; 4.96)	2.36; 5	48	0.242^a^
*More than 2 minimum wages*	4.86 (4.57; 4.96)	3.86; 5	28	
Employment status				
*Unemployed*	4.79 (4.43; 4.93)	3.93; 5	30	
*Formal/informal employment*	4.79 (4.43; 5)	3.86; 5	19	0.832^b^
*Government assistance*	4.86 (4.57; 5)	2.36; 5	27	
Transport cost to hospital				
*Up to $ 2*	4.79 (4.21; 4.93)	2.36; 5	26	
*$ 2–4*	4.82 (4.64; 4.93)	4.07; 5	20	0.762^b^
*$ 4–10*	4.79 (4.14; 5)	3.79; 5	17	
*More than $ 10*	4.86 (4.50; 5)	3.64; 5	13	
Travel Time to Hospital				
*< 30 minutes*	4.79 (4.43; 4.93)	3.93; 5	17	
*30–45 minutes*	4.57 (4.43; 4.79)	3.64; 5	13	
*45 minutes–1 hour*	4.93 (4.71; 5)	3.79; 5	14	0.287^b^
*1–1.5 hours*	4.86 (4.43; 4.96)	3.86; 5	12	
*> 1.5 hours*	4.75 (4.32; 4.96)	2.36; 5	20	

Q1, first quartile; Q3, third quartile; n, number of patients;

^a^ Mann–Whitney test;

^b^ Kruskal–Wallis test.

## DISCUSSION

This study evaluated the sociodemographic profile of patients, the feasibility of teleoncology implementation, and patient satisfaction with remote care in a socially vulnerable population of patients with breast cancer treated within the public health system. The findings indicate that telemedicine can be incorporated into routine breast cancer care with high patient satisfaction and meaningful practical benefits, including reduced travel-related costs and less time spent attending consultations. Notably, 83% of patients reported that they would recommend telemedicine to others.

To our knowledge, this is the first prospective study conducted in Brazil outside the pandemic setting to examine telemedicine implementation in the clinical management of patients with breast cancer while also providing a sociodemographic characterization of the population and a structured evaluation of teleconsultation quality using a validated instrument.

The sociodemographic characteristics of this cohort are consistent with those reported for predominantly low-income patients with breast cancer in Brazil, a population that often faces substantial economic and social challenges.^19^ This population often relies on the public healthcare system and experiences barriers to accessing oncology care, including transportation difficulties, limited financial resources, and competing priorities, such as work and family responsibilities.^20,21^

A central aim of this study was to evaluate the potential advantages of remote care, including convenience, safety, and effectiveness in meeting the needs of each clinical case. In particular, the study examined whether teleoncology could help overcome barriers associated with in-person visits, including transportation demands, financial costs, time constraints, and work absenteeism, all of which are particularly relevant for socioeconomically vulnerable patients undergoing breast cancer treatment.

The findings underscore the burden imposed by in-person care in this population. Most patients (66.7%) reported a monthly family income of up to two minimum wages, 57% relied on public transportation, 35.5% spent up to $ 2 to reach the hospital, and 28% required over 90 minutes of travel time. These data illustrate the logistical and financial challenges associated with routine face-to-face consultations. In this context, the studies support the role of telemedicine in reducing both direct and indirect costs related to cancer care, including transportation expenses, lodging, and income lost because of missed work.^22^

This practical benefit was also reflected in our findings. Among patients with formal or informal employment, 38.1% reported avoiding the loss of a workday because of teleconsultation. This suggests that remote care may meaningfully affect both financial stability and daily functioning in this vulnerable population. In addition, a substantial proportion of patients (38.7%) depended on government assistance, including welfare or disability benefits, further emphasizing the economic fragility of the study population and the value of care models that reduce the burden of in-person visits.

Contrary to the assumption that socially vulnerable populations may have limited access to digital technologies, most patients in this cohort reported having the basic means required to participate in teleconsultations, including internet access and mobile phones. These findings suggest that technological access may be less restrictive than often presumed, even in low-income settings. Previous studies similarly indicate that telemedicine can be a feasible and effective approach for delivering oncology care to vulnerable populations.^20,21,23,24^

Most patients reported a positive experience with teleconsultations, achieving an NPS score of 79.8%, which is classified as excellent. Other studies suggest that the average NPS for in-person healthcare typically falls between 60% and 80%, while telehealth appointments generally range from 70% to 90%.^25,26^ Furthermore, repeated use of teleconsultations did not lead to a decline in patient satisfaction, and some patients even reported increased satisfaction over time.^24^ Thus, the high satisfaction scores observed in this study suggest that teleconsultation can be a viable and well-received approach for delivering oncology care to predominantly low-income patients with breast cancer.

Additionally, most patients were satisfied with the telehealth experience, with a median TSQ score of 4.8 out of 5. These findings are consistent with previous reports showing high patient satisfaction with telehealth, particularly regarding convenience, reduced travel time, and improved access to care.^24^ Other studies have reported similarly high satisfaction with teleoncology, particularly during the COVID-19 pandemic when in-person visits were restricted.^21,23^ Patient satisfaction remained high even after multiple teleconsultations, with no significant differences in satisfaction scores between the first and second appointments.

Reported drivers of telehealth satisfaction commonly include convenience, lower cost, ease of use, effective communication, and elimination of travel time,^27,27^ all of which align with the experience reported by patients in the present study.

Importantly, repeated exposure to teleconsultation did not appear to reduce patient satisfaction. Satisfaction scores remained high across successive consultations, and some patients reported higher scores after subsequent remote visits. Although no significant difference was observed between the first and second teleconsultations, these findings suggest that teleoncology remains acceptable over time for patients appropriately selected for remote follow-up.^24^

At the same time, telemedicine was not universally accepted. Among clinically eligible patients, only 51.1% completed teleconsultation, and patient preference for in-person care was the most common reason for non-completion. Inability to establish contact also contributed to non-completion in a smaller proportion of cases. Compared with a prior study conducted by our group during the COVID-19 pandemic, uptake in the present study was lower (51.1% vs. 71%),^21^ suggesting that social isolation may have increased acceptance of remote care during the pandemic period.

Sonagli et al. conducted a retrospective study at a tertiary cancer center in São Paulo during the COVID-19 pandemic, and showed that telemedicine was adopted to mitigate an almost 50% reduction in outpatient visits and was feasible for follow-up breast cancer, screening, and selected benign breast conditions.^28^

The preference for in-person visits among a substantial subgroup of clinically eligible patients indicates that telemedicine is not universally accepted, even when remote care is clinically appropriate. This finding underscores the need for patient education regarding the scope and benefits of teleconsultation, while ensuring that clinical management is not compromised. Refusal of teleconsultation may also reflect diverse barriers, including the perceived value of physical examination, communication preferences, privacy concerns, mistrust of remote care, technological constraints, or perceived clinical seriousness. Future studies should incorporate targeted surveys of patients who decline teleconsultation to better characterize these barriers and inform patient-centered implementation strategies.

Collectively, teleoncology can be a viable option for delivering care to predominantly low-income and low-literacy patients with breast cancer, particularly when the aim is to reduce barriers related to travel, time, cost, and work absenteeism. However, its role is best understood within a selective and clinically appropriate model of care in which telemedicine complements, rather than replaces, in-person consultations.

This study has some limitations. It was conducted at a single institution within a specific geographic region, which may limit the generalizability of the findings. The absence of a control group receiving in-person care also restricted the ability to compare outcomes more rigorously. Although the sample of 93 patients provided important insight into patient characteristics and experiences with teleoncology, a larger sample might have offered greater statistical power to detect subtler associations across various outcome measures. Additionally, the lack of access to video-based consultations at our institution may have influenced the quality of the consultations.

Future studies should examine the long-term effects of teleoncology on clinical outcomes, treatment adherence, and overall quality of life among socioeconomically vulnerable patients with breast cancer. Further studies are warranted to better understand the barriers and facilitators influencing teleoncology adoption in this population and to identify strategies for sustainable implementation and scale-up within public health systems.

## CONCLUSION

This study shows that teleoncology is a feasible and acceptable strategy for follow-up care in a clinically selected subgroup of predominantly low-income patients with breast cancer. Among patients considered eligible for teleconsultation, more than half completed the remote visit, indicating meaningful uptake in an appropriate clinical context. Although a substantial proportion of eligible patients preferred in-person care, these findings support integrating telemedicine alongside conventional consultations to reduce sociodemographic barriers and promote more equitable access to breast cancer management.

## Data Availability

The datasets generated and/or analyzed during the current study are not publicly available due to patient privacy and institutional restrictions but are available from the corresponding author upon reasonable request.
